# Spunlace Cotton Nonwoven-Reinforced Sodium Alginate/Bamboo Charcoal Photothermal Hydrogel Evaporator for Solar-Driven Water Purification

**DOI:** 10.3390/gels12100899

**Published:** 2026-10-04

**Authors:** Haifeng Zhang, Guangfu Chen, Hao He, Xia Zhu, Tonggang Liu

**Affiliations:** 1 School of Mechatronic Engineering, China University of Mining and Technology, Xuzhou 221116, China; zhanghf@ntu.edu.cn; 2 School of Textile and Clothing, Nantong University, Nantong 226019, China

**Keywords:** solar interfacial evaporation, spunlace cotton nonwoven, sodium alginate hydrogel, bamboo charcoal, seawater desalination

## Abstract

Solar-driven interfacial evaporation offers a low-energy and sustainable route for seawater desalination and the purification of complex water matrices. However, freestanding hydrogel evaporators often suffer from insufficient mechanical robustness and interfacial salt accumulation under high-salinity conditions. Herein, a spunlace cotton nonwoven/sodium alginate/bamboo charcoal (SC/SA/BC) composite interfacial evaporator was fabricated through a facile impregnation–ionic crosslinking strategy, using a spunlace cotton nonwoven (SC) as the reinforcing scaffold, sodium alginate (SA) as the hydrophilic hydrogel matrix, and bamboo charcoal powder (BC) as the photothermal component. The Ca^2+^-crosslinked SA network immobilized the bamboo charcoal particles and formed continuous hydrated mass-transport pathways, while the spunlace fibrous network provided capillary water-supply channels and robust mechanical support. Consequently, SC/SA/BC exhibited substantially improved mechanical performance compared with the freestanding SA/BC hydrogel. Under 1 sun irradiation using a 3.5 wt% NaCl solution, SC/SA/BC achieved an evaporation rate of approximately 1.80 kg m^−2^ h^−1^ and maintained stable performance during 8 h of continuous operation. The evaporation–condensation process markedly reduced the concentrations of major ions in natural seawater and effectively retained nonvolatile dyes. Through the synergistic integration of a spunlace fibrous scaffold, a hydrated hydrogel network, and a bamboo-charcoal-based photothermal component, the proposed evaporator simultaneously achieved mechanical reinforcement, sustained water supply, efficient photothermal conversion, and salt-tolerant operation. This work provides a promising strategy for constructing structurally stable and readily fabricated solar interfacial evaporation materials.

## 1. Introduction

Freshwater scarcity and water pollution have emerged as major challenges to global sustainable development [1]. Although seawater and brackish water represent abundant alternative water resources, conventional thermal desalination processes are generally energy-intensive, whereas reverse osmosis relies on a continuous electricity supply, high-pressure equipment, and regular membrane maintenance, thereby limiting its deployment in islands, remote regions, and emergency water-supply scenarios. Solar-driven interfacial evaporation localizes solar absorption and heat generation at the water–air interface, minimizing ineffective heating of the bulk water and offering a promising low-energy route for seawater desalination and wastewater purification [2,3,4,5]. A high-performance solar interfacial evaporator requires not only broadband solar absorption and efficient photothermal conversion, but also sustained water supply, minimized heat loss [6,7], unobstructed vapor escape, regulated salt-ion transport [8,9], and long-term structural stability [10,11]. Hydrogels, which contain abundant hydrophilic functional groups and three-dimensional hydrated networks, have been widely employed in interfacial evaporators because of their capabilities for water storage, water transport [12,13], and immobilization of photothermal components [14]. However, freestanding hydrogels often suffer from insufficient mechanical strength, structural fragility, and deformation caused by swelling or dehydration-induced shrinkage [15,16]. These limitations may compromise their handling stability and the integrity of internal water-transport pathways, thereby restricting their sustained operation and potential for large-area applications.

Fibrous materials are attractive platforms for solar evaporation because of their flexibility, high porosity, and interconnected capillary pathways, enabling them to function simultaneously as water-transport media and supporting substrates for photothermal functional layers [17,18]. In recent years, cotton fabrics, carbon-fiber fabrics, and nonwoven materials have been used to construct solar evaporators [19,20], while wettability gradients [21], Janus architectures [22,23], and fabric–hydrogel composites have been developed to improve water supply and regulate salt transport [24,25]. Unlike woven fabrics with regularly arranged warp and weft yarns, spunlace nonwovens consist of randomly oriented fibers entangled by high-pressure water jets to form a three-dimensional porous network. This structure provides multidirectional capillary water-transport pathways, abundant interfacial area for loading functional components, and good flexibility. The hydroentangled structure of a spunlace cotton nonwoven provides mechanical integrity and flexibility, while the intrinsic hydrophilicity of cotton fibers and interconnected inter-fiber pores facilitate capillary water transport. These combined characteristics make a spunlace cotton nonwoven a suitable scaffold for integrating hydrogel reinforcement and continuous water supply within a single composite architecture.

Sodium alginate is a naturally derived polysaccharide rich in hydroxyl and carboxylate groups. It can form hydrophilic three-dimensional networks through Ca^2+^-mediated ionic crosslinking and offers several advantages, including aqueous processability, mild gelation conditions, and good compatibility with porous substrates [26]. Bamboo charcoal powder possesses a dark aromatic carbon structure, broad-spectrum solar absorption [27,28], and relatively low material cost, making it a promising biomass-derived photothermal material [29,30]. However, direct incorporation of bamboo charcoal powder often leads to particle aggregation and migration, whereas freestanding sodium alginate hydrogels generally exhibit limited mechanical stability. Therefore, immobilizing bamboo charcoal particles within a Ca^2+^-crosslinked sodium alginate network while employing a spunlace nonwoven for structural reinforcement and continuous capillary water supply may enable the synergistic integration of photothermal conversion, hydrophilic mass transport, and mechanical support.

Herein, a spunlace cotton nonwoven-reinforced sodium alginate/bamboo charcoal (SC/SA/BC) photothermal hydrogel evaporator was developed through a simple aqueous impregnation and Ca^2+^-mediated ionic crosslinking strategy. The hydroentangled cotton scaffold provides mechanical support and interconnected capillary water-supply pathways, while the hydrated SA/BC phase integrates photothermal conversion and water transport. Through the integration of these complementary components, the proposed architecture aims to address the mechanical limitations of freestanding hydrogels while maintaining effective solar interfacial evaporation. The contributions of the individual components were evaluated through comparative structural characterization, mechanical testing, and evaporation measurements. This work demonstrates a straightforward approach to integrating mechanical reinforcement and solar evaporation functionality within a hydrogel–fiber composite architecture.

## 2. Results and Discussion

### 2.1. Construction and Working Mechanism of the SC/SA/BC Composite Evaporator

**Figure 1:**
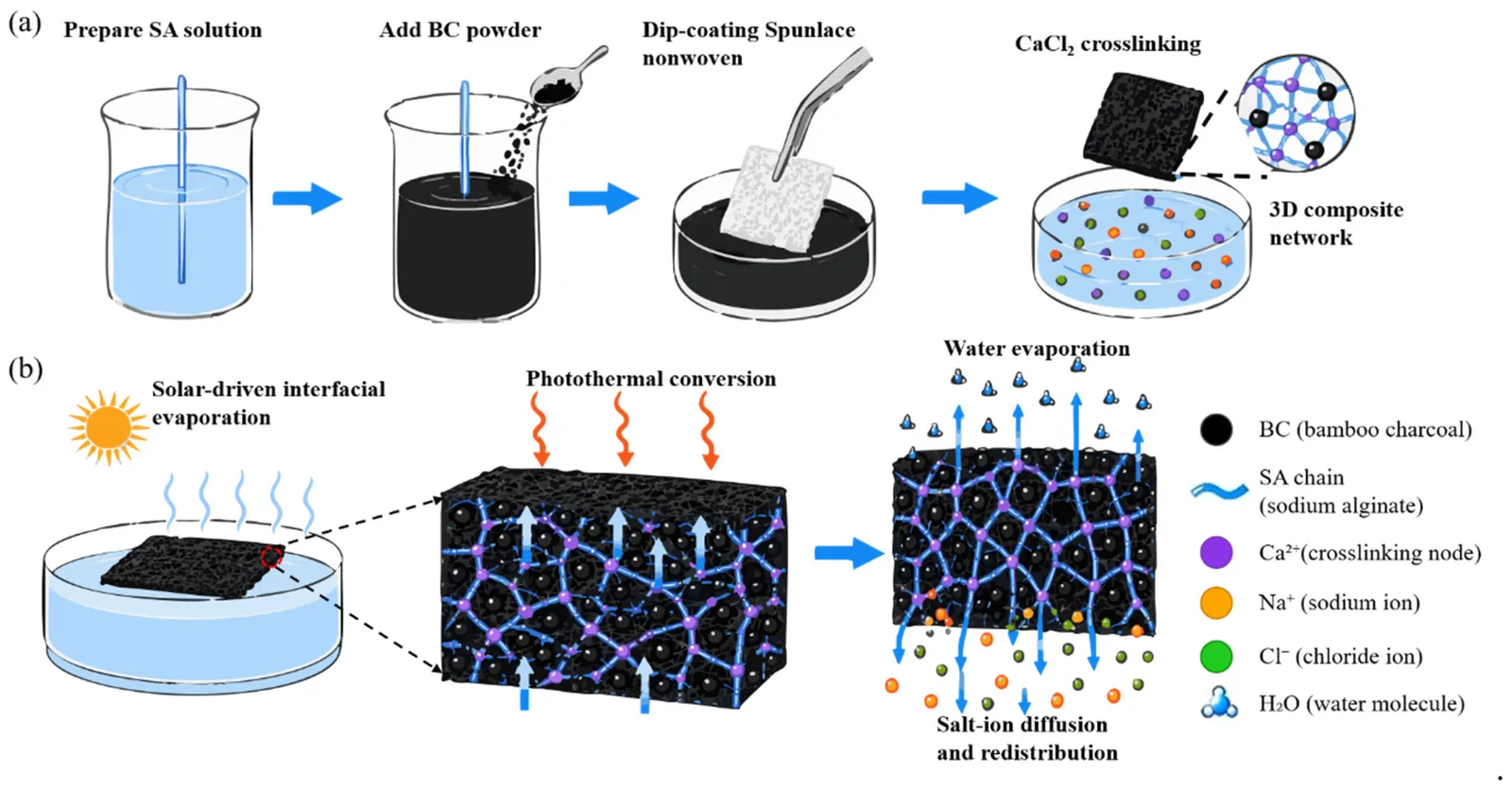
Schematic illustration of the fabrication process and proposed working mechanism of the SC/SA/BC composite evaporator. (**a**) Fabrication process of SC/SA/BC. (**b**) Mechanisms of solar-driven interfacial evaporation and salt-ion transport.

To achieve efficient photothermal conversion, continuous water supply, and structural stability, an SC/SA/BC composite interfacial evaporator was constructed using a spunlace nonwoven as the porous supporting scaffold, a Ca^2+^-crosslinked sodium alginate hydrogel as the hydrophilic mass-transport network, and bamboo charcoal powder as the photothermal component. As illustrated in Figure 1a, the SA/BC precursor infiltrated and adhered to the spunlace fibrous network through capillary action, followed by CaCl_2_-induced ionic crosslinking. Ca^2+^ ions coordinated with the carboxylate groups of sodium alginate to form a crosslinked network, thereby immobilizing the bamboo charcoal particles within the hydrogel and fibrous scaffold. This process ultimately produced an integrated composite structure consisting of spunlace fibers, calcium alginate hydrogel, and photothermal bamboo charcoal particles.

The solar-driven interfacial evaporation mechanism of SC/SA/BC is illustrated in Figure 1b. Under solar irradiation, the bamboo charcoal particles distributed on the surface and throughout the interior of the composite rapidly absorb incident light and convert it into heat [31]. The generated thermal energy is preferentially localized at the upper water–air interface, thereby promoting the conversion of interfacial water into vapor. Meanwhile, bulk water is continuously transported toward the evaporation interface through the combined action of the capillary pathways within the spunlace fibrous network and the hydrophilic sodium alginate hydrogel network, compensating for evaporative water loss and preventing local dehydration [32]. This synergistic process of photothermal conversion, capillary water supply, and interfacial evaporation facilitates continuous and stable vapor generation. During saline water evaporation, water molecules escape from the evaporation interface, whereas nonvolatile ions such as Na^+^ and Cl^−^ remain in the liquid phase. The interconnected hydrophilic network not only sustains water replenishment but also facilitates the diffusion and redistribution of salt ions along the concentration gradient toward regions of lower concentration, thereby alleviating excessive salt accumulation at the evaporation interface and suppressing the formation of a continuous, dense salt layer. This structural design helps address the insufficient mechanical strength and poor handling stability of freestanding hydrogels while taking advantage of the porous water-transport characteristics of the spunlace fibrous network, thereby providing a robust structural basis for efficient photothermal response, high-performance interfacial evaporation, and salt-tolerant operation.

### 2.2. Structural Characteristics and Fundamental Properties of SC/SA/BC

**Figure 2:**
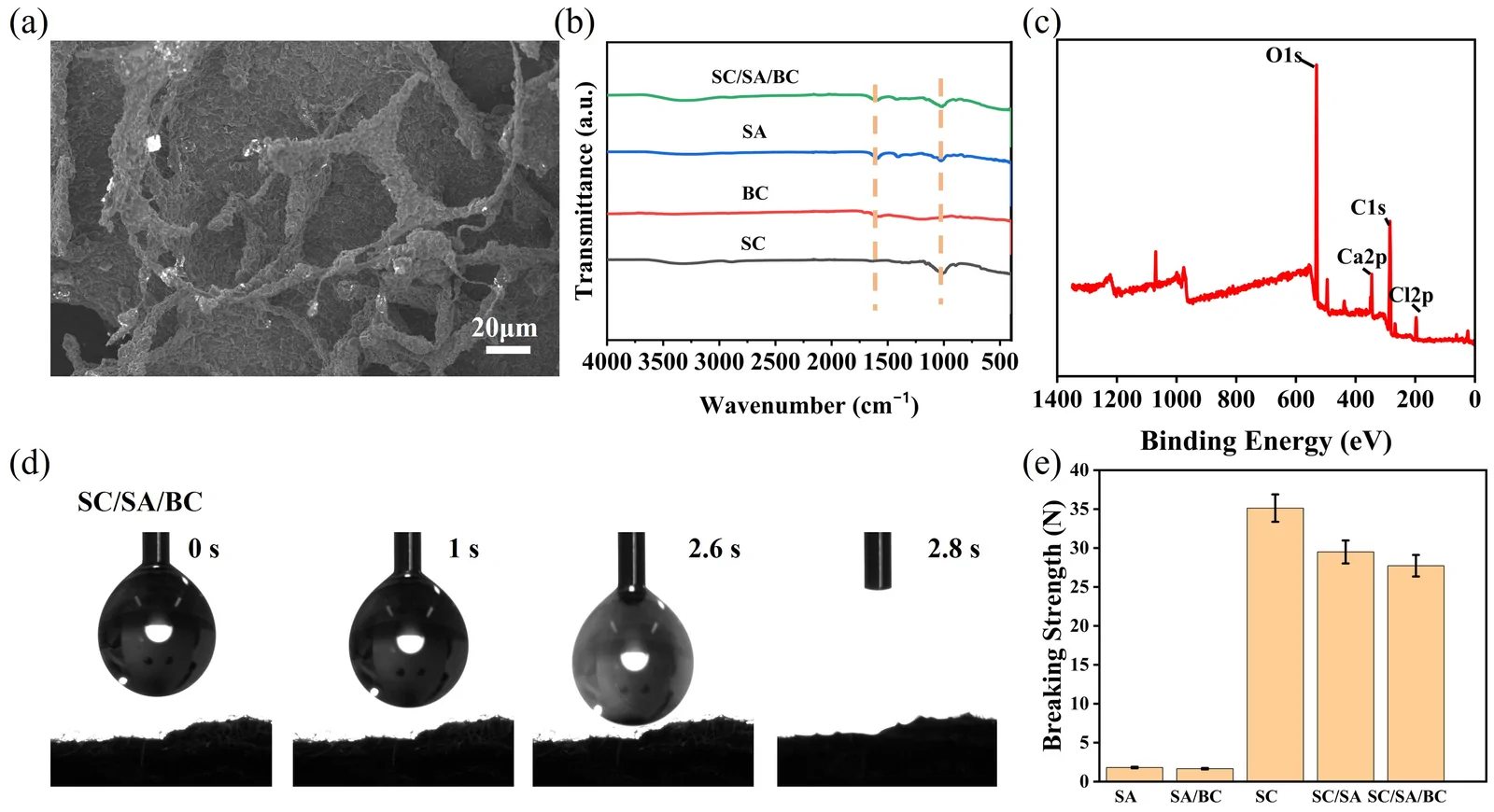
Structural characterization and fundamental properties of SC/SA/BC. (**a**) SEM image. (**b**) FTIR spectra. (**c**) XPS survey spectrum. (**d**) Sequential photographs showing the dynamic water-absorption process at 0, 1, 2.6, and 2.8 s. (**e**) Breaking forces of different samples.

The structural characteristics and fundamental properties of SC/SA/BC were first investigated. As shown in Figure S1, SC/SA/BC is a black, square-shaped, sheet-like composite with relatively continuous dark surface coverage and a flexible fibrous structure. After CaCl_2_ crosslinking, the SA/BC coating remained attached to the spunlace cotton scaffold, forming an intact composite evaporator. As shown in Figure 2a, SC/SA/BC exhibited a typical three-dimensional interconnected fibrous network. The fiber surfaces were continuously coated with a rough hydrogel/bamboo charcoal composite layer, which formed bridging structures at some fiber junctions. Despite the surface coating, the original open pores and interconnected channels were largely preserved. This hierarchical composite architecture enabled the spunlace fibrous scaffold to provide stable mechanical support for the hydrogel while maintaining continuous pathways for capillary water transport and vapor escape.

The Fourier transform infrared spectra of samples with different compositions are presented in Figure 2b. SC, SA, BC, and SC/SA/BC exhibited characteristic absorption bands corresponding to their respective components. The SC/SA/BC composite retained the major characteristic bands of its constituent materials. The bands at approximately 1596 and 1420 cm^−1^ were assigned to the asymmetric and symmetric stretching vibrations of COO^−^, respectively. Their changes relative to those of pristine SA were consistent with Ca^2+^–carboxylate coordination.

XPS analysis was further performed to examine the surface elemental composition of SC/SA/BC. As shown in Figure 2c, the XPS survey spectrum exhibited pronounced C 1s and O 1s signals, together with a characteristic Ca 2p peak, confirming the successful incorporation of Ca into the composite system. Combined with the shifts in the COO^−^ absorption bands observed by FTIR, these results suggest that Ca^2+^ participated in the ionic crosslinking of adjacent sodium alginate chains, thereby facilitating the formation of a stable hydrogel network. Collectively, the SEM, FTIR, and XPS results, together with the macroscopic observations in Figure S1, support the formation of a Ca^2+^-crosslinked SA/BC gel phase immobilized within the spunlace cotton fibrous scaffold.

Rapid wetting and sustained water supply are essential for the stable operation of solar-driven interfacial evaporation. The dynamic water-absorption behavior of SC/SA/BC is illustrated in Figure 2d. As shown in the sequential photographs recorded at 0, 1, 2.6, and 2.8 s, the water droplet progressively spread across the material surface and was gradually absorbed, becoming no longer visible at approximately 2.8 s. This observation demonstrates the good wettability and water-absorption capability of the composite evaporator. Dynamic wetting tests of the control samples (Figure S2) revealed varying degrees of rapid water absorption, indicating that both the spunlace fibrous network and the hydrophilic sodium alginate structure facilitate water transport.

The breaking forces of the different samples were further compared to evaluate the reinforcing effect of the spunlace nonwoven scaffold on the hydrogel. As shown in Figure 2e, the freestanding SA and SA/BC hydrogels exhibited breaking forces of only approximately 1–2 N, indicating that the unsupported hydrogels possessed insufficient mechanical robustness for practical handling and continuous operation. In contrast, pristine SC exhibited a breaking force of approximately 35 N, demonstrating its favorable load-bearing capability. After integration with the spunlace nonwoven, SC/SA and SC/SA/BC retained breaking forces of approximately 29 and 28 N, respectively, both substantially higher than those of the freestanding SA and SA/BC hydrogels. These results demonstrate that the spunlace nonwoven scaffold markedly improves the structural integrity and handling stability of the hydrogel while preserving a relatively high load-bearing capacity in the resulting composites [33].

### 2.3. Solar Absorption and Photothermal Conversion Performance

**Figure 3:**
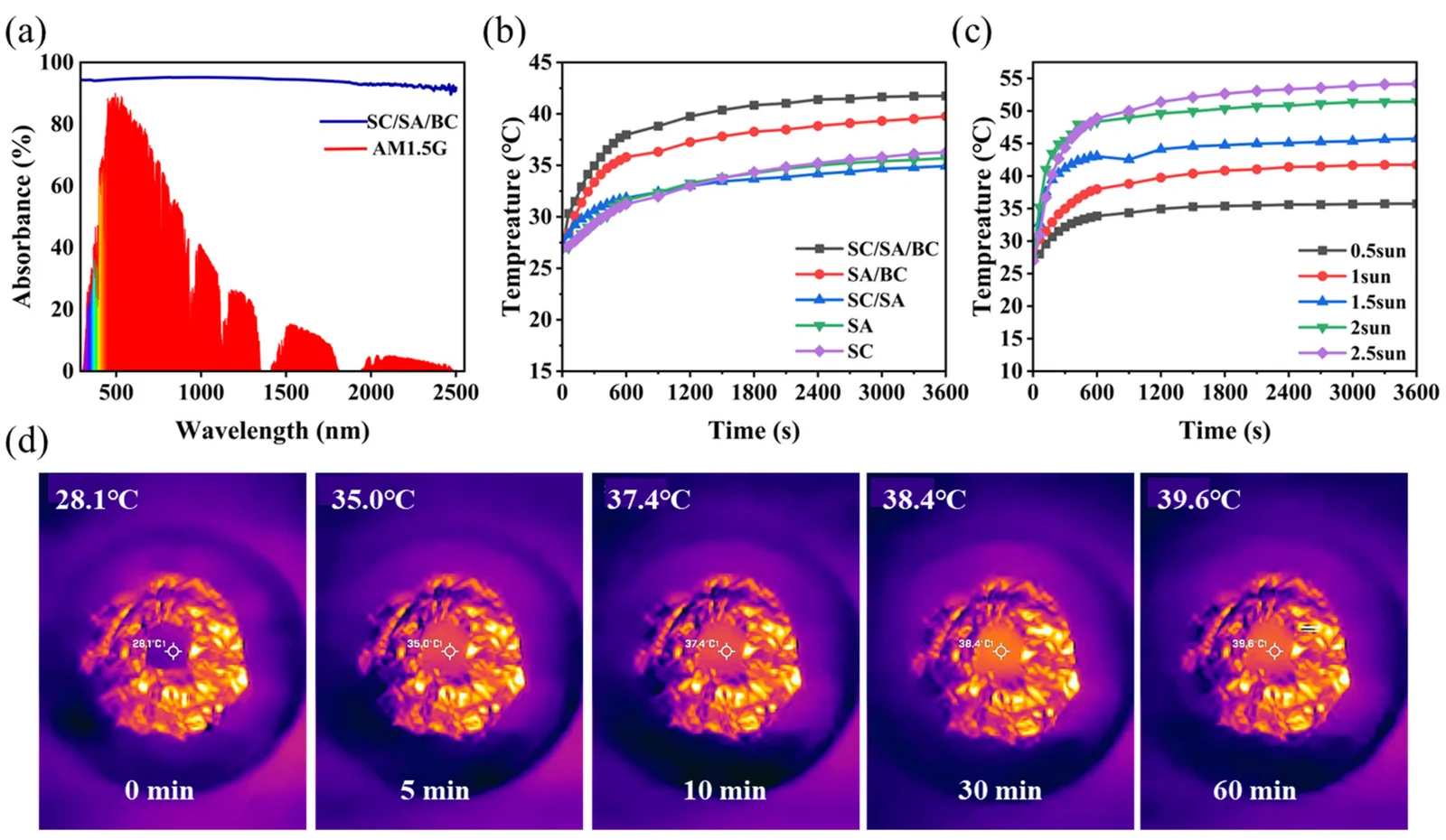
Solar absorption and photothermal performance of SC/SA/BC. (**a**) Solar spectral absorptance. (**b**) Photothermal heating profiles of different samples. (**c**) Temperature evolution of SC/SA/BC under different irradiation intensities. (**d**) Infrared thermal images of SC/SA/BC.

Solar harvesting and photothermal conversion are critical determinants of the energy-utilization efficiency and evaporation performance of interfacial evaporators. To evaluate the photothermal response of the SC/SA/BC composite evaporator, its spectral absorption characteristics and the surface-temperature evolution of samples with different compositions under simulated solar irradiation were investigated. As shown in Figure 3a, SC/SA/BC exhibited excellent broadband solar absorption over the wavelength range of 300–2500 nm, with an absorptance exceeding 90% across most of the solar spectrum. This strong light-harvesting capability was primarily attributed to the bamboo charcoal powder. In addition, the multiscale rough and porous architecture may enhance internal light scattering and multiple reflections, thereby extending the optical path length and further improving solar energy utilization.

The temperature profiles of the different samples under 1 sun irradiation are presented in Figure 3b. Upon illumination, the temperature of each sample increased rapidly, followed by a progressively slower rise until a quasi-steady state was reached. Among all samples, SC/SA/BC exhibited the most pronounced photothermal response, with its surface temperature increasing from approximately 28 °C to approximately 42 °C, markedly higher than those of the control samples. The surface temperatures of SC/SA/BC and SA/BC were substantially higher than those of the bamboo-charcoal-free samples, indicating that bamboo charcoal was the primary functional component responsible for efficient solar absorption and photothermal conversion within the composite system.

The temperature response of SC/SA/BC under different irradiation intensities was further investigated. As shown in Figure 3c, the surface temperature of the sample increased progressively as the irradiation intensity was raised from 0.5 to 2.5 sun. The corresponding steady-state temperatures under 0.5, 1.0, 1.5, 2.0, and 2.5 sun were approximately 36, 42, 45, 51, and 54 °C, respectively. Under all irradiation intensities, SC/SA/BC exhibited a rapid initial temperature rise followed by gradual stabilization as thermal equilibrium was approached. The monotonic increase in steady-state temperature with incident light intensity demonstrates that SC/SA/BC can rapidly respond to varying solar irradiance and exhibits good irradiance adaptability and tunable photothermal conversion behavior.

Infrared thermography further visualized the evolution of the surface temperature distributions of the different samples under 1 sun irradiation. As shown in Figure 3d and Figures S3–S7, the surface temperature of SC/SA/BC rapidly reached 35.0 °C after 5 min of irradiation, after which the high-temperature region progressively expanded and stabilized. Its temperature rise was markedly greater than those of the control samples and was consistent with the temperature evolution trend shown in Figure 3b. These results further confirm that bamboo charcoal is the principal component responsible for efficient photothermal conversion, while the spunlace scaffold and hydrogel network contribute to a stable overall photothermal response.

### 2.4. Solar-Driven Interfacial Evaporation Performance

**Figure 4:**
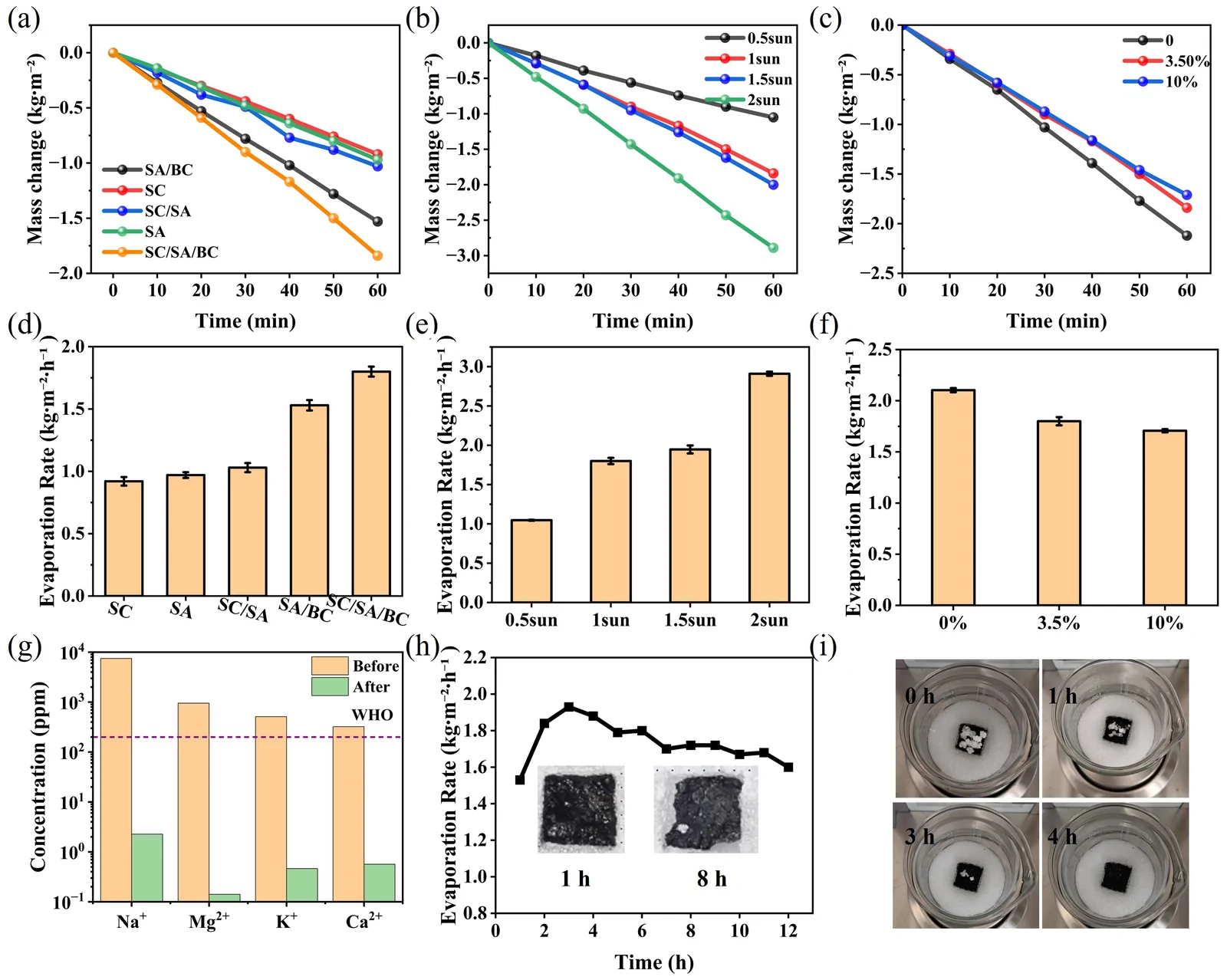
Solar evaporation, desalination, and salt-tolerance performance of SC/SA/BC. (**a**) Mass changes of different samples during evaporation. (**b**) Mass changes of SC/SA/BC under different irradiation intensities. (**c**) Mass changes of SC/SA/BC at different salt concentrations; (**d**–**f**) Evaporation rates corresponding to panels (**a**–**c**), respectively. (**g**) Concentrations of major ions in the original seawater and the corresponding condensate. (**h**) Continuous evaporation stability. (**i**) Redissolution of surface-deposited salt crystals.

The solar-driven interfacial evaporation behavior of the different samples was first compared under identical irradiation conditions. As shown in Figure 4a, the mass per unit area of all samples decreased approximately linearly with irradiation time. Among them, SC/SA/BC exhibited the greatest mass loss, reaching approximately 1.80 kg m^−2^ after 60 min of irradiation, substantially higher than those of the other samples. As shown in Figure 4d, the evaporation rates of SC, SA, and SC/SA were approximately 0.92, 0.97, and 1.03 kg m^−2^ h^−1^, respectively. After the incorporation of bamboo charcoal, the evaporation rate increased to approximately 1.53 kg m^−2^ h^−1^ for SA/BC and further reached approximately 1.80 kg m^−2^ h^−1^ for SC/SA/BC. These results indicate that the broadband solar absorption and photothermal conversion capabilities of bamboo charcoal play a key role in enhancing evaporation performance. Meanwhile, the spunlace nonwoven scaffold provides continuous capillary water-transport pathways and stable structural support, while the sodium alginate hydrogel forms a water-rich hydrophilic network. The synergistic integration of these three components ensures sustained water replenishment and thermal energy input at the evaporation interface [34].

The effect of solar irradiance on the evaporation behavior of SC/SA/BC was further investigated. As shown in Figure 4b, the mass loss per unit area increased continuously with time over the irradiation range of 0.5–2 sun and became more pronounced as the irradiation intensity increased. As shown in Figure 4e, increasing the irradiation intensity from 0.5 to 2 sun increased the evaporation rate of SC/SA/BC from approximately 1.03 to 2.90 kg m^−2^ h^−1^. The continuous increase in evaporation rate with incident solar power demonstrates that SC/SA/BC can effectively respond to variations in solar irradiance and convert the additional incident energy into the thermal energy required for interfacial water evaporation, indicating good adaptability to different irradiation intensities.

To evaluate the evaporation performance of SC/SA/BC in saline water, its evaporation behavior was investigated at different NaCl concentrations. As shown in Figure 4c, the pure water system exhibited the greatest mass loss, whereas the mass loss per unit area progressively decreased as the NaCl concentration increased from 0 to 3.5 and 10 wt%. Correspondingly, the evaporation rates of SC/SA/BC in pure water and 3.5 and 10 wt% NaCl solutions were approximately 2.10, 1.80, and 1.72 kg m^−2^ h^−1^, respectively (Figure 4f). Although increasing salinity suppressed evaporation to some extent, SC/SA/BC still maintained an evaporation rate above 1.7 kg m^−2^ h^−1^ in 10 wt% NaCl solution, indicating that its porous hydrophilic network can sustain relatively stable water transport and interfacial evaporation over a broad salinity range. To further evaluate the evaporation performance of SC/SA/BC, a comparison with representative solar interfacial evaporators reported in the literature was conducted, as summarized in Table S1. The comparison indicates that the present study integrates mechanical support from the fibrous scaffold, continuous water supply through the hydrophilic network, and photothermal conversion through a simple impregnation–ionic crosslinking process, while achieving comparable evaporation performance under different salinity conditions. This design highlights the advantages of SC/SA/BC in terms of fabrication simplicity, structural support, and the integration of saline-water evaporation functionality.

To assess the practical desalination and salt-tolerance performance of SC/SA/BC, natural seawater purification, continuous evaporation, and salt-redissolution tests were further conducted. As shown in Figure 4g, the concentrations of Na^+^, Mg^2+^, K^+^, and Ca^2+^ in the condensate decreased by several orders of magnitude relative to those in the original seawater, demonstrating effective retention of the major nonvolatile salt ions during the evaporation–condensation process. As shown in Figure 4h and Figure S8, SC/SA/BC maintained a stable evaporation rate of approximately 1.72–1.86 kg m^−2^ h^−1^ during 8 h of continuous irradiation, without forming a continuous, dense salt layer covering the evaporation interface. To further evaluate its continuous-operation performance, the evaporation test was extended to 12 h (Figure S9). Although the evaporation rate gradually decreased during the later stage, SC/SA/BC sustained evaporation throughout the test, and no continuous, dense salt layer was observed on the evaporation surface, as shown in the corresponding hourly photographs. Furthermore, salt crystals predeposited on the sample surface gradually redissolved upon contact with the bulk water and almost completely disappeared within approximately 4.5 h (Figure 4i and Figure S10). These results indicate that the continuous hydrophilic pathways formed by the spunlace fibrous network and sodium alginate hydrogel sustain water replenishment and facilitate salt redissolution and redistribution [35], thereby mitigating interfacial salt accumulation and maintaining stable saline water evaporation [36]. Thus, the observed salt tolerance is attributed to sustained water replenishment and passive salt redistribution within the integrated fibrous–hydrogel structure, rather than to the action of any single component alone.

### 2.5. Purification of Complex Water Matrices and Outdoor Solar Evaporation Performance

**Figure 5:**
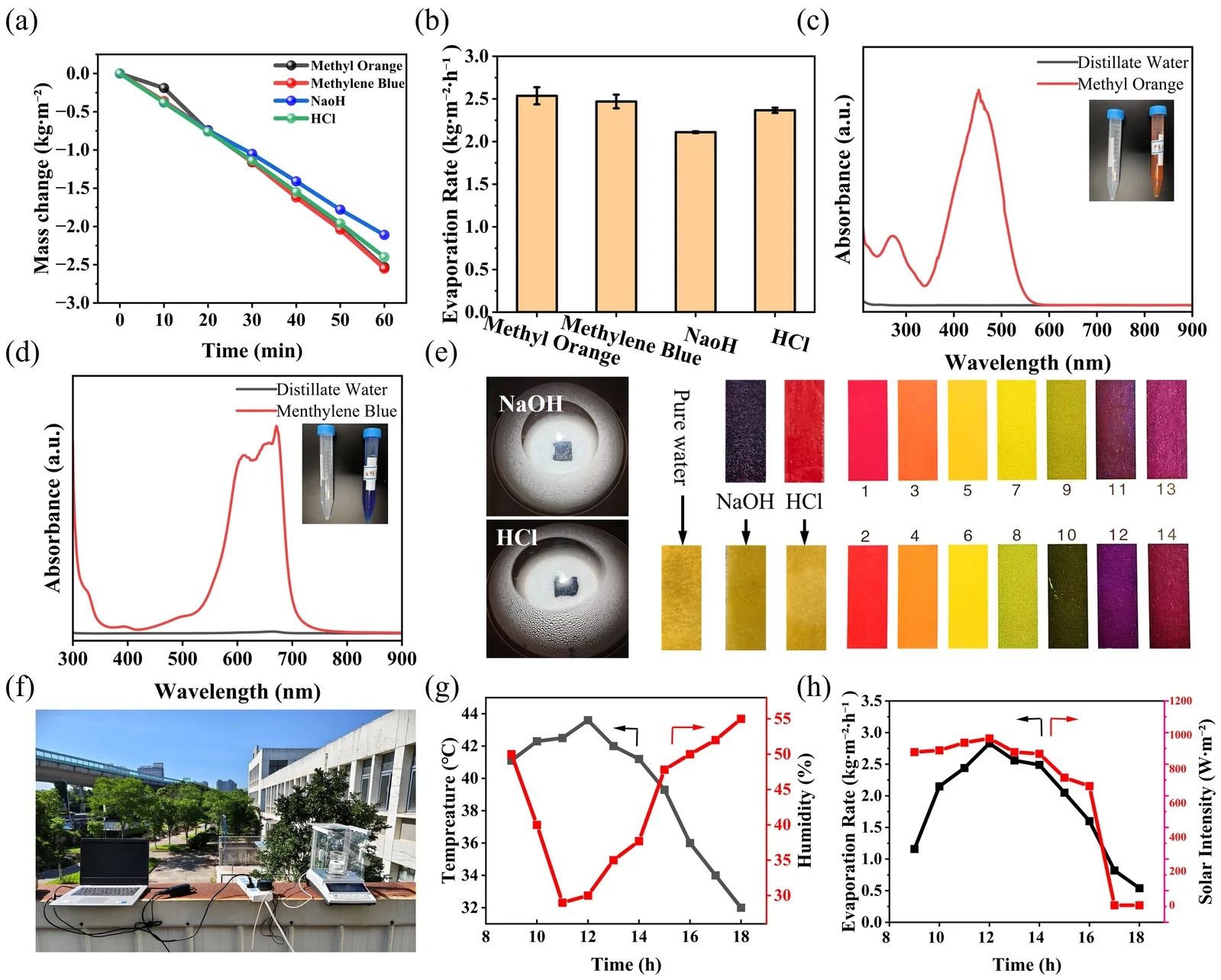
Complex water purification and outdoor evaporation performance of SC/SA/BC. (**a**) Mass changes during the evaporation of different model water matrices. (**b**) Corresponding evaporation rates. (**c**,**d**) UV–vis spectra of methyl orange and methylene blue feed solutions and their corresponding condensates. (**e**) Preliminary pH tests of condensates obtained from different model water matrices. (**f**) Outdoor evaporation setup. (**g**) Ambient temperature and relative humidity during the outdoor test. (**h**) Solar irradiance and outdoor evaporation rate during the outdoor test.

To evaluate the applicability of SC/SA/BC to complex water matrices, methyl orange, methylene blue, NaOH, and HCl solutions were selected as model feedwaters for evaporation tests. As shown in Figure 5a, the cumulative mass loss of each system increased approximately linearly with irradiation time, indicating that SC/SA/BC maintained stable interfacial evaporation under different water conditions. The evaporation rates in methyl orange and methylene blue solutions were approximately 2.53 and 2.47 kg m^−2^ h^−1^, respectively, whereas slightly lower values were obtained in the NaOH and HCl solutions (Figure 5b). The differences in evaporation rate may be associated with variations in solution composition, ionic content, and optical absorption. Despite the differences in water composition, SC/SA/BC exhibited consistently stable evaporation performance, demonstrating good adaptability to complex water matrices.

The retention of dyes during the evaporation–condensation process was further evaluated using UV–vis spectroscopy. As shown in Figure 5c,d, the methyl orange and methylene blue feed solutions exhibited distinct characteristic absorption peaks at approximately 460 and 660–670 nm, respectively, whereas the corresponding peaks were nearly absent from the condensates, which appeared colorless and transparent. These results indicate that the nonvolatile dyes were effectively retained in the feed solutions [37,38]. Meanwhile, SC/SA/BC maintained its overall structural integrity and sustained evaporation in both NaOH and HCl solutions. Preliminary pH-indicator-paper measurements showed that the indicator colors of the resulting condensates shifted toward the neutral range (Figure 5e), suggesting that the composite evaporator possesses a certain degree of tolerance to acidic and alkaline environments.

The outdoor evaporation performance of SC/SA/BC is presented in Figure 5f–h. During the test, the solar irradiance reached approximately 1000 W m^−2^ at around 12:00, while the instantaneous evaporation rate of SC/SA/BC simultaneously increased from approximately 1.1 kg m^−2^ h^−1^ at 09:00 to approximately 2.8 kg m^−2^ h^−1^. Thereafter, the evaporation rate gradually decreased with declining solar irradiance, reaching approximately 0.5 kg m^−2^ h^−1^ at 18:00. The evaporation rate generally followed the temporal variation in solar irradiance and was also influenced by the ambient temperature and relative humidity. These results demonstrate that SC/SA/BC maintained continuous evaporation and responded dynamically to variations in natural solar irradiance under outdoor conditions [39,40].

## 3. Conclusions

In this study, a spunlace nonwoven/sodium alginate/bamboo charcoal composite interfacial evaporator (SC/SA/BC) was fabricated through an impregnation–Ca^2+^-mediated ionic crosslinking strategy. The spunlace nonwoven scaffold provided mechanical support and capillary water-transport pathways, while the crosslinked sodium alginate hydrogel immobilized the bamboo charcoal particles and established a hydrophilic mass-transport network. Meanwhile, the bamboo charcoal powder endowed the composite with excellent broadband solar absorption and photothermal conversion capabilities. The synergistic integration of these three components enabled rapid wetting, good mechanical integrity, and stable interfacial water supply.

SC/SA/BC exhibited an absorptance exceeding 90% across most of the solar spectrum and achieved an evaporation rate of approximately 1.80 kg m^−2^ h^−1^ under 1 sun irradiation, while maintaining a relatively stable evaporation rate during 8 h of continuous operation without a sustained decline. The composite also facilitated the redissolution and redistribution of surface-deposited salt crystals, markedly reduced the concentrations of major salt ions and nonvolatile dyes in the condensate, and exhibited good responsiveness to variations in solar irradiance under outdoor conditions. This spunlace nonwoven reinforcement strategy provides a viable route for developing structurally stable and readily fabricated materials for solar-driven seawater desalination and the purification of complex water matrices.

## 4. Materials and Methods

### 4.1. Materials

Sodium alginate powder (SA, analytical reagent grade, product No. S100126, nominal viscosity of 200 ± 20 mPa·s) and calcium chloride (CaCl_2_, analytical reagent grade) were purchased from Shanghai Aladdin Biochemical Technology Co., Ltd. (Shanghai, China). Bamboo charcoal powder (BC, food grade) was supplied by Wenzhou Minglan Trading Co., Ltd. (Wenzhou, Zhejiang, China). Sodium chloride (NaCl, analytical reagent grade) and methyl orange (MO, indicator grade) were obtained from Sinopharm Chemical Reagent Co., Ltd. (Shanghai, China). Methylene blue (MB, indicator grade) was purchased from Beijing Solarbio Science & Technology Co., Ltd. (Beijing, China). Universal pH indicator paper (pH 1–14) was supplied by Shanghai 3A Reagent Co., Ltd. (Shanghai, China). Hydrochloric acid (HCl, analytical reagent grade) and sodium hydroxide (NaOH, analytical reagent grade) were obtained from Shanghai Runjie Technology Development Co., Ltd. (Shanghai, China) and Xilong Scientific Co., Ltd. (Shantou, Guangdong, China), respectively. The spunlace nonwoven, composed of 100% cotton fibers with a basis weight of 35 g m^−2^, was provided by Jianghua New Materials Technology Co., Ltd. (Nantong, Jiangsu, China). Natural seawater was collected from the Bohai Sea.

### 4.2. Preparation of the SA and SA/BC Precursor Solutions

Sodium alginate powder (2.00 g) was added to 98 mL of deionized water and magnetically stirred at 80 °C for 1 h until a homogeneous and transparent 2 wt% SA solution was obtained. The resulting solution was allowed to cool naturally to room temperature and then left undisturbed for 2 h to remove air bubbles generated during stirring. Subsequently, 0.20 g of bamboo charcoal powder was added to 10 mL of the as-prepared 2 wt% SA solution. The mixture was magnetically stirred at room temperature for 30 min to uniformly disperse the bamboo charcoal particles, yielding the SA/BC precursor solution. This formulation was selected to provide a hydrophilic polymer matrix for hydrogel formation while incorporating bamboo charcoal as the photothermal component.

### 4.3. Fabrication of the Composite Evaporator

Before use, the spunlace cotton nonwoven was rinsed with deionized water and dried to a constant mass. The dried nonwoven was completely immersed in the SA/BC precursor solution for 1 min to allow sufficient wetting and infiltration of the precursor into the fibrous pores. After removal from the solution, excess and nonuniformly distributed precursor on the sample surface was gently removed using a glass rod or scraper. Impregnation was performed before ionic crosslinking to allow the SA/BC precursor to infiltrate the fibrous network prior to hydrogel formation. The impregnated sample was subsequently immersed in a 0.5 wt% CaCl_2_ aqueous solution for 5 min to induce ionic crosslinking. During this process, Ca^2+^ ions interacted with the carboxylate groups of the SA chains, thereby crosslinking the polymer network and immobilizing the SA/BC hydrogel within the spunlace fibrous scaffold. The resulting composite evaporator was denoted as SC/SA/BC. After crosslinking, the samples were rinsed three times with deionized water to remove residual CaCl_2_ and unbound components, and were then refrigerated at 4 °C until further use. The SC/SA, SA, and SA/BC control samples were prepared using analogous procedures. For SC/SA, bamboo charcoal powder was omitted from the impregnation solution, whereas the SA and SA/BC controls were prepared without the spunlace nonwoven scaffold.

### 4.4. Characterization

The surface and cross-sectional morphologies of the samples were examined using a desktop scanning electron microscope (SEM, EM-30 PLUS, COXEM, Daejeon, Republic of Korea). Prior to observation, the samples were sputter-coated with gold to improve electrical conductivity, and the accelerating voltage was set to 10 kV. Dynamic wetting behavior was characterized at room temperature using a contact-angle goniometer (SDC-350, Kunshan Shengding, Kunshan, Jiangsu, China), with a 5 μL deionized-water droplet as the probe liquid; the spreading and absorption of the droplet on the sample surface were recorded. Tensile properties were measured in accordance with GB/T 3923.1-2013 using a geotextile universal strength tester (WDW-10, Jinan Sida, Jinan, Shandong, China). The specimens were 100 mm × 20 mm in size, with a gauge length of 80 mm and a crosshead speed of 100 mm min^−1^. At least three replicate specimens were tested for each sample. The chemical structures of the samples were analyzed by attenuated total reflectance Fourier transform infrared spectroscopy (ATR-FTIR, Nicolet iS50, Thermo Fisher Scientific, Waltham, MA, USA) using a diamond ATR crystal over the wavenumber range of 4000–500 cm^−1^. Diffuse reflectance and transmittance spectra were recorded over 300–2500 nm using a UV–vis–NIR spectrophotometer equipped with an integrating sphere (UV-3600, Shimadzu, Kyoto, Japan), with a BaSO_4_ standard white plate used as the reflectance reference. The surface elemental composition of SC/SA/BC was analyzed by X-ray photoelectron spectroscopy (XPS, K-Alpha, Thermo Fisher Scientific, Waltham, MA, USA) using monochromatic Al Kα radiation, with a pass energy of 200 eV for the survey scan. The photothermal response was characterized using a thermocouple temperature recorder (TA612C, Suzhou TASI, Suzhou, Jiangsu, China) and an infrared thermal imaging camera (SE-187, FLIR Systems, Wilsonville, OR, USA). Temperature–time profiles were continuously recorded at 1 s intervals using the thermocouple, while infrared thermography was performed on independent specimens to obtain representative surface-temperature distributions at different irradiation times.

### 4.5. Photothermal Evaporation and Water Purification Tests

A CME-SL1000 xenon lamp (Beijing China Education Au-Light, Beijing, China) was used to simulate solar irradiation, and the incident intensity was calibrated using an SM206 PLUS solar power meter (Xinbao Instruments, Shenzhen, China). Samples with an effective evaporation area of 4.0 cm^2^ were placed in a thermally insulated evaporation setup containing 100 mL of the test solution. The mass change was recorded at 1 min intervals using an electronic analytical balance (PTY-A220, Fujian Huazhi, Putian, Fujian, China). Unless otherwise specified, indoor experiments were conducted at 25 ± 2 °C and a relative humidity of 50 ± 5%, and each test was repeated at least three times, except for the outdoor experiment. The standard evaporation test was performed using 3.5 wt% NaCl solution under 1 sun irradiation for 60 min. The effect of irradiation intensity was evaluated in 3.5 wt% NaCl solution under 0.5, 1.0, 1.5, and 2.0 sun, whereas the effect of salinity was investigated under 1 sun using 0, 3.5, and 10 wt% NaCl solutions. For the continuous stability test, 3.5 wt% NaCl solution was used as the water source, and the evaporator was operated under 1 sun for 8 h; the evaporation rate and surface salt deposition state were recorded at 1 h intervals. For the salt redissolution test, 0.50 g of NaCl crystals was placed on the surface of a fully wetted sample, with its lower surface in contact with 3.5 wt% NaCl solution, and the evolution of the salt crystals was recorded over 0–4.5 h under dark conditions. For natural seawater desalination, the generated water vapor was condensed and collected, and the concentrations of major ions in the original seawater and condensate were measured by inductively coupled plasma mass spectrometry (ICP-MS, NexION 350X, PerkinElmer, Shelton, CT, USA). Methyl orange, methylene blue, HCl, and NaOH solutions were used as model complex water matrices. The dye feed solutions and corresponding condensates were analyzed by UV–vis spectroscopy, while the condensates obtained from the acidic and alkaline model solutions were preliminarily evaluated using universal pH indicator paper. The outdoor experiment was conducted from 09:00 to 18:00 on 10 July 2026 at Nantong University, Nantong, China, with the mass change, solar irradiance, ambient temperature, and relative humidity recorded simultaneously.

The net evaporation rate after subtraction of dark evaporation was calculated according to Equation (1):

(1) $$v=\frac{{\Delta m}_{light}-{\Delta m}_{dark}}{A\cdot \Delta t}$$

where *v* is the net evaporation rate (kg m^−2^ h^−1^), Δ*m*_light_ and Δ*m*_dark_ are the mass losses measured under illuminated and dark conditions, respectively (kg), *A* is the effective evaporation area (m^2^), and Δ*t* is the evaporation time (h).

## Data Availability

The original contributions presented in this study are included in the article/Supplementary Materials. Further inquiries can be directed to the corresponding author.

## Supplementary Materials

The following supporting information can be downloaded at: https://www.mdpi.com/article/10.3390/gels12100899/s1. Figure S1: Macroscopic photographs of SC/SA/BC before and after CaCl^−^ crosslinking, and the resulting composite evaporator; Figure S2: Dynamic wetting behavior of different samples at 0 and 10 ms; Figure S3: Infrared thermal images of SC/SA/BC at different irradiation times under 1 sun; Figure S4: Infrared thermal images of SA/BC at different irradiation times under 1 sun; Figure S5: Infrared thermal images of SC/SA at different irradiation times under 1 sun; Figure S6: Infrared thermal images of SC at different irradiation times under 1 sun; Figure S7: Infrared thermal images of SA at different irradiation times under 1 sun; Figure S8: Surface salt deposition on SC/SA/BC during 8 h of continuous saline-water evaporation; Figure S9: Continuous evaporation performance of the SC/SA/BC evaporator in 3.5 wt% NaCl solution under 1 sun irradiation over 12 h: evaporation rates and corresponding hourly photographs of the evaporator; Figure S10: Redissolution of salt crystals deposited on the surface of SC/SA/BC during static standing; Table S1: Comparison of the evaporation performance of representative solar interfacial evaporators.
