# Supplementary material for: Spunlace Cotton Nonwoven-Reinforced Sodium Alginate/Bamboo Charcoal Photothermal Hydrogel Evaporator for Solar-Driven Water Purification
Source: Gels. 2026 Oct 4;12(10):899. doi: 10.3390/gels12100899 (PMC13704654; doi:10.3390/gels12100899)
Supplement: Supplementary file 1 [file gels-12-00899-s001.zip › gels-4537931-supplementary.pdf]

Supplementary Material

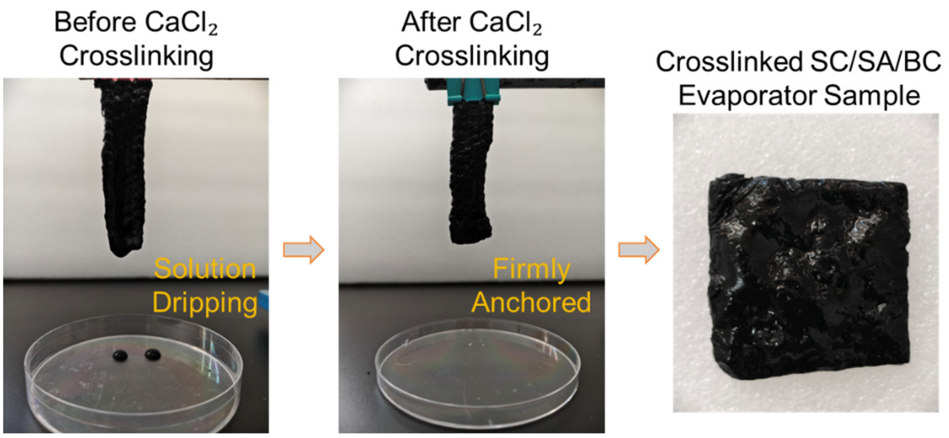

Figure S1. Macroscopic photographs of SC/SA/BC before and after $CaCl_2$ crosslinking, and the resulting composite evaporator.

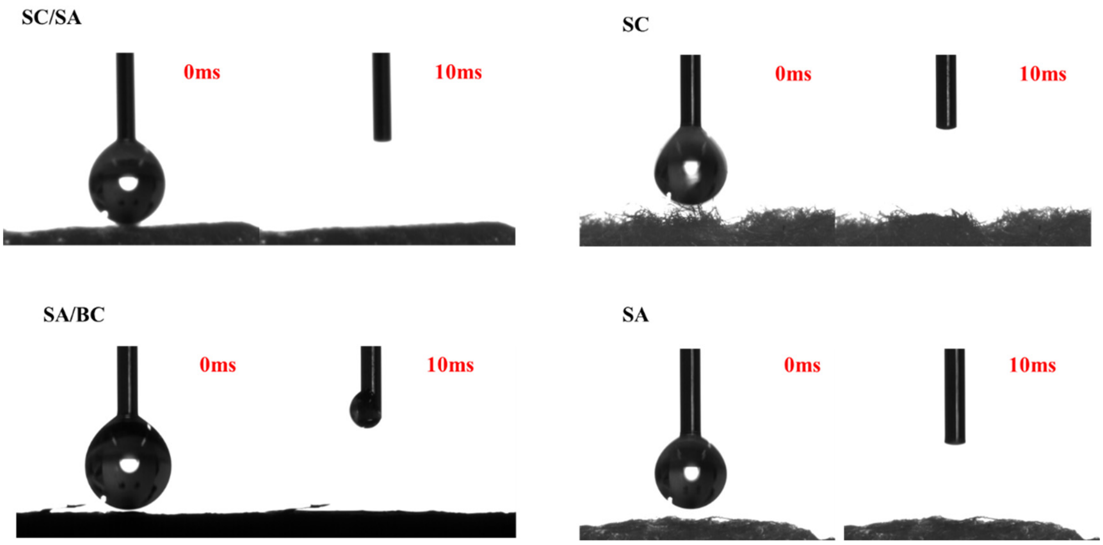

Figure S2. Dynamic wetting behavior of different samples at 0 and 10 ms.

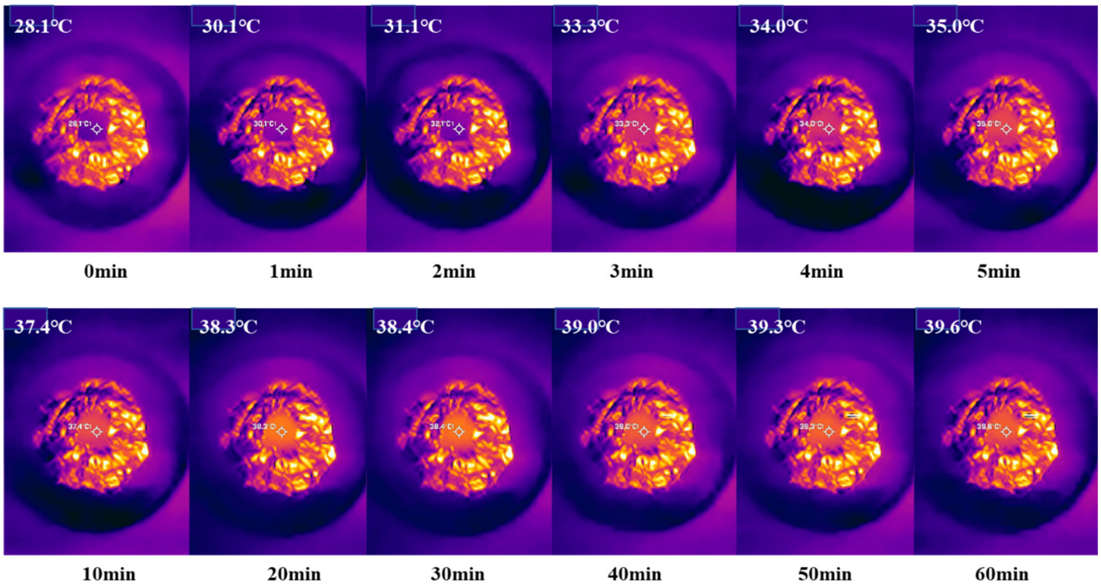

Figure S3. Infrared thermal images of SC/SA/BC at different irradiation times under 1 sun.

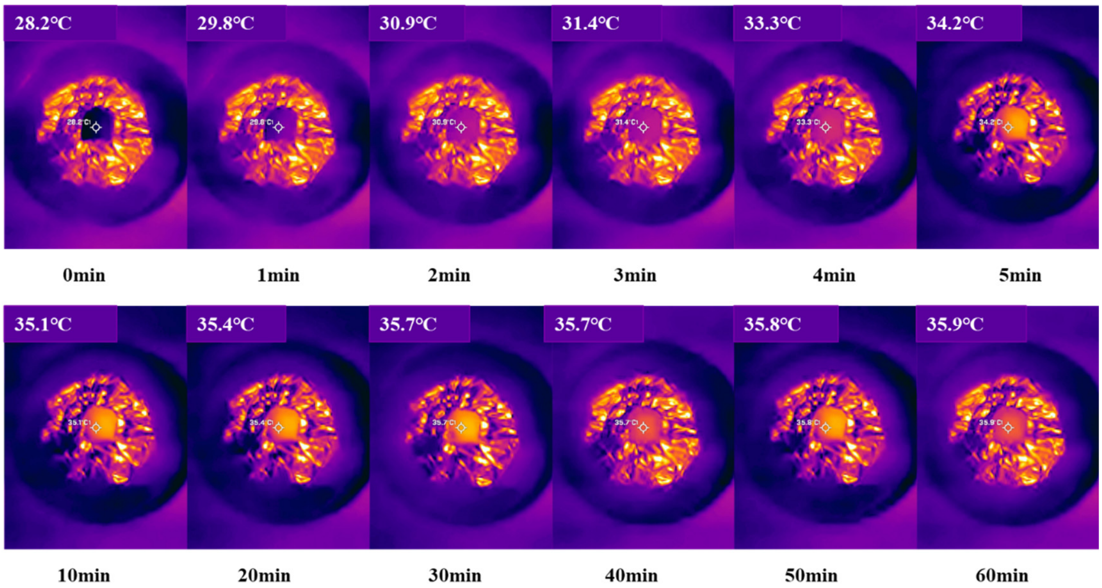

Figure S4. Infrared thermal images of SA/BC at different irradiation times under 1 sun.

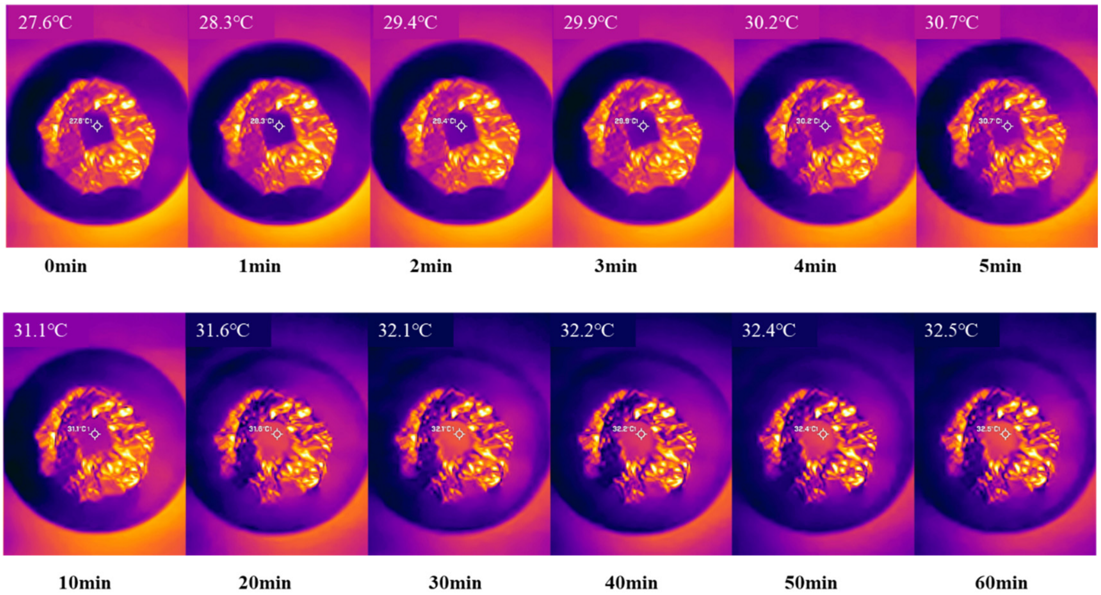

Figure S5. Infrared thermal images of SC/SA at different irradiation times under 1 sun.

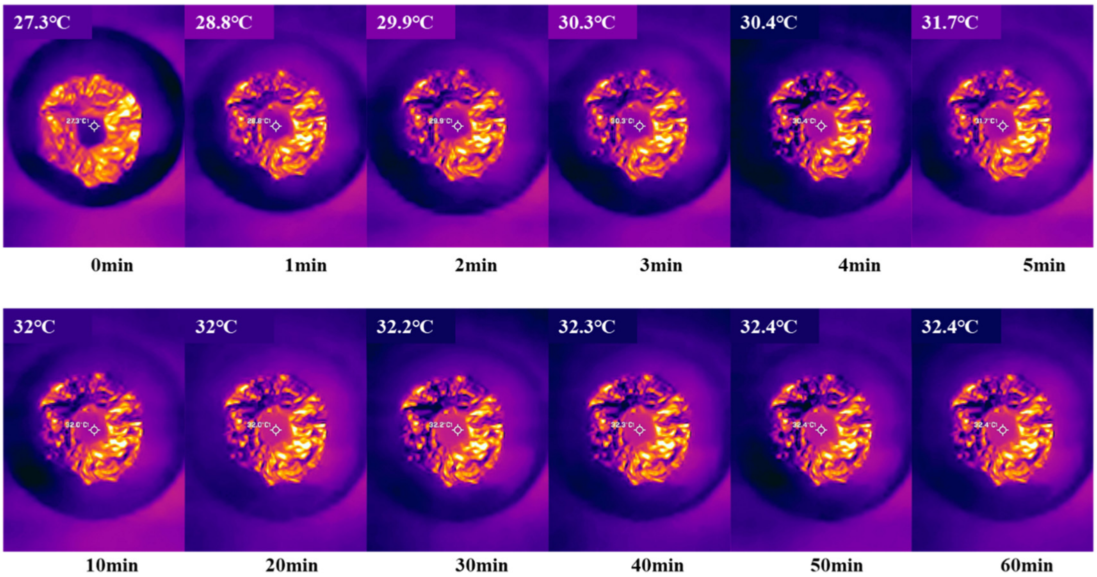

Figure S6. Infrared thermal images of SC at different irradiation times under 1 sun.

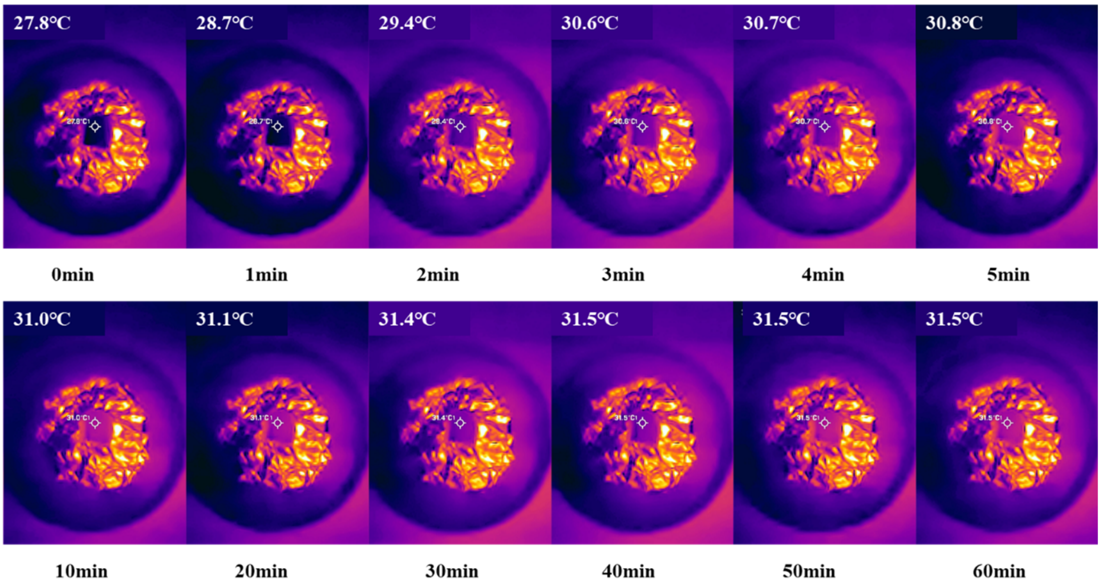

Figure S7. Infrared thermal images of SA at different irradiation times under 1 sun.

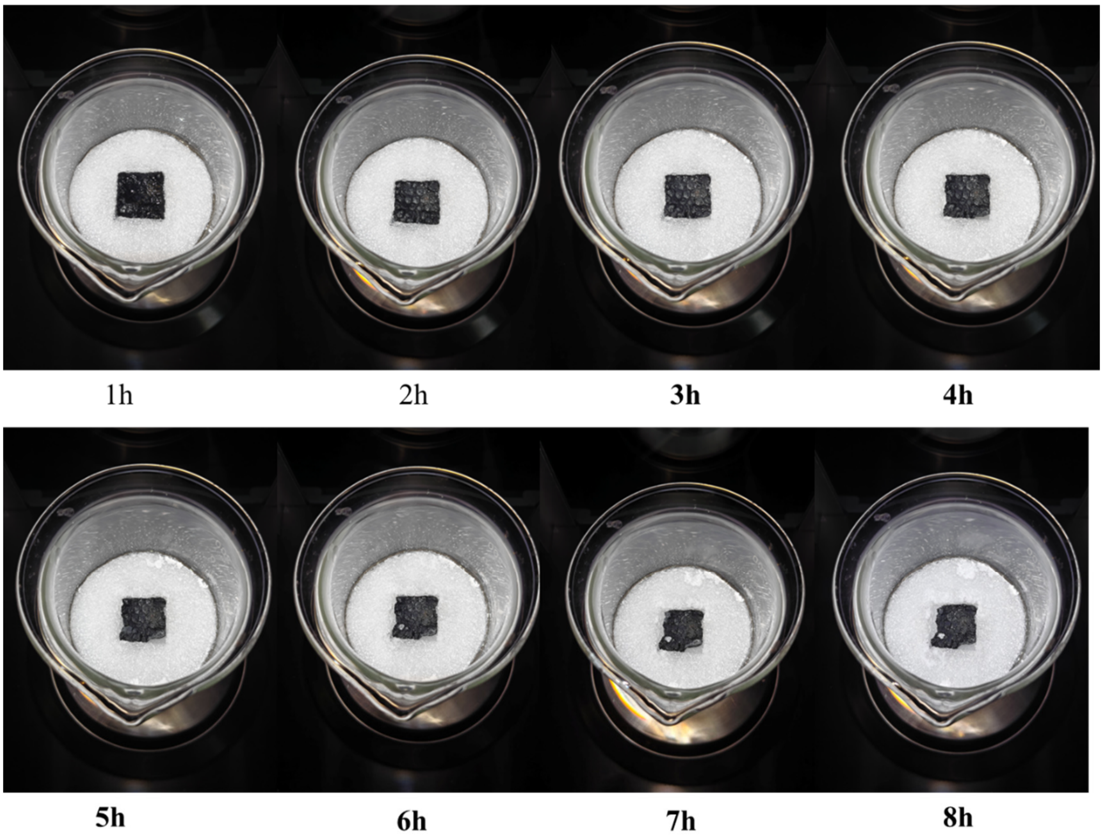

Figure S8. Surface salt deposition on SC/SA/BC during 8 h of continuous saline-water evaporation.

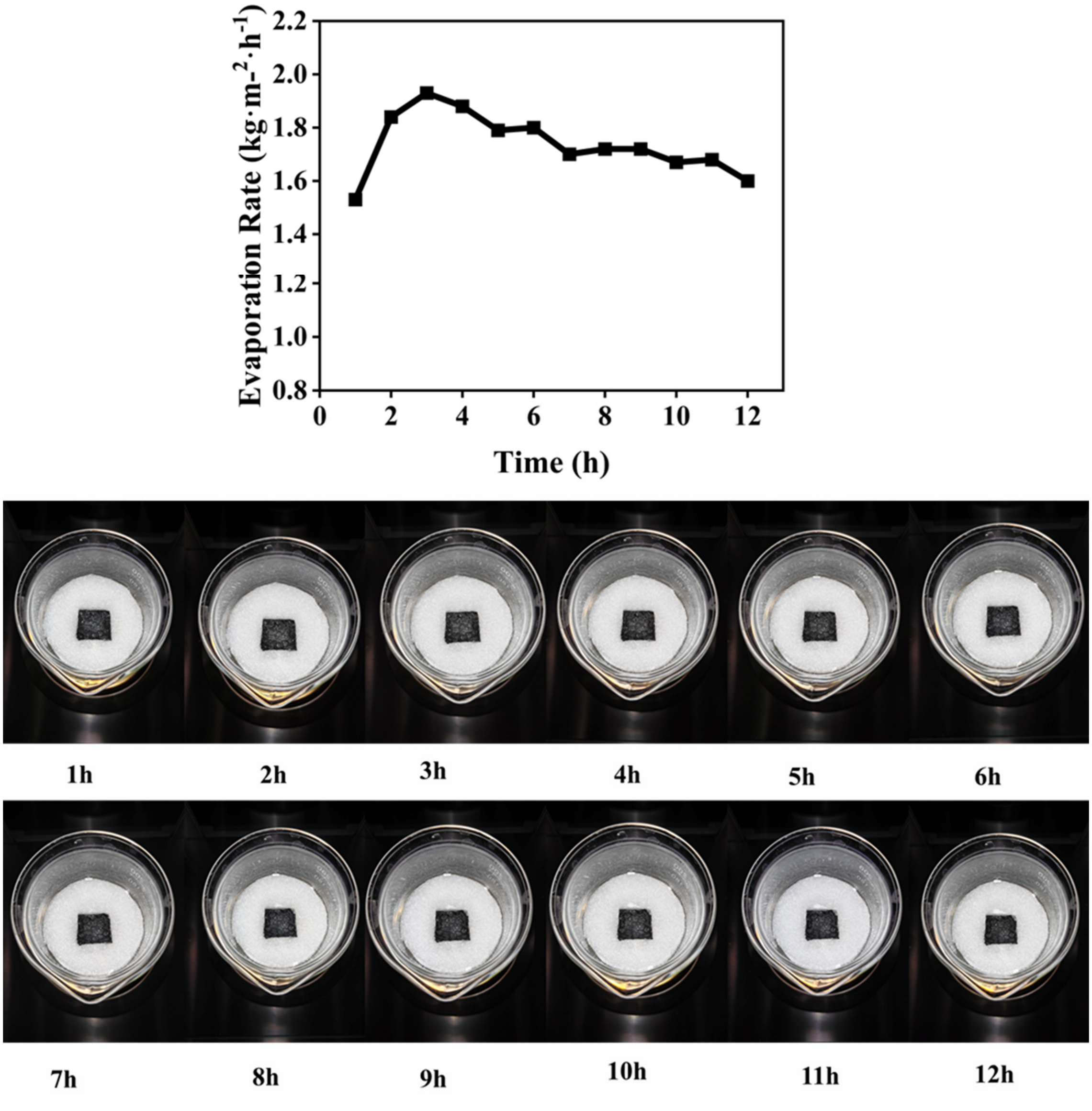

Figure S9. Continuous evaporation performance of the SC/SA/BC evaporator in 3.5 wt% NaCl solution under 1 sun irradiation over 12 h: evaporation rates and corresponding hourly photographs of the evaporator.

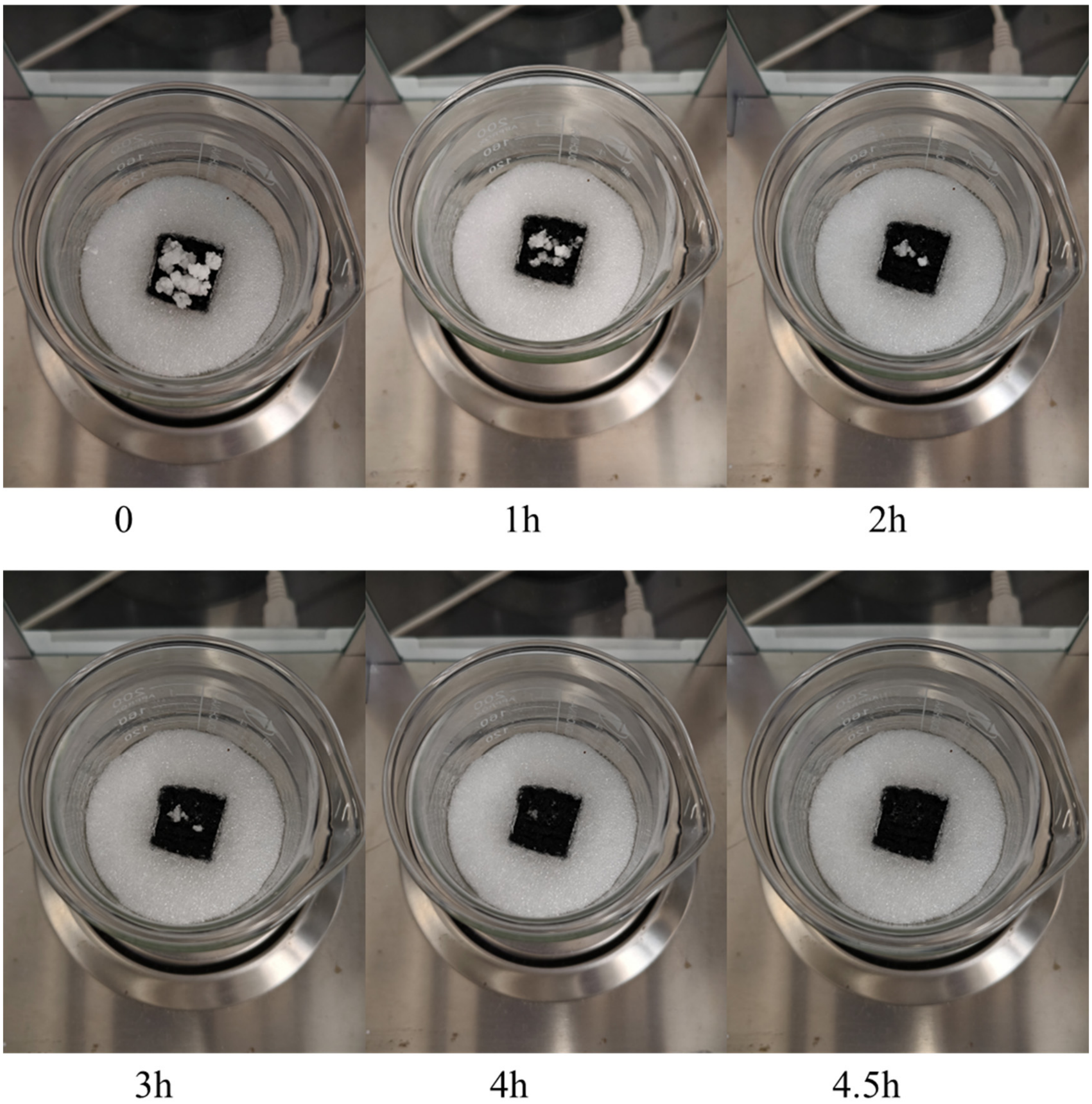

Figure S10. Redissolution of salt crystals deposited on the surface of SC/SA/BC during static standing.

Table S1. Comparison of the evaporation performance of representative solar interfacial evaporators.

**Evaporator / reference**	**Feedwater**	**Evaporation rate ($kg\ m^{-2}\ h^{-1}$)**	**Relevant feature**
Alginate/carbon-black-coated ramie fabric [17]	Water	1.81	Draped configuration with tunable water supply
PDA/carbon-black/PP nonwoven [18]	Seawater	1.68	One-way water-supply configuration
Janus hydrogel–fabric [22]	Pure water; 3.5 wt% NaCl	1.60; 1.55	Salt-management design
MoS_2-based membrane [38]	10 wt% NaCl	1.71	Integrated evaporation and water purification
SC/SA/BC (this study)	Pure water	2.10	Simple impregnation – ionic crosslinking process
SC/SA/BC (this study)	3.5 wt% NaCl	1.80	Fibrous reinforcement with a breaking force of approximately 28 N
SC/SA/BC (this study)	10 wt% NaCl	1.72	Hydrophilic network for continuous water supply
